# A bibliometric analysis of RNA methylation in diabetes mellitus and its complications from 2002 to 2022

**DOI:** 10.3389/fendo.2022.997034

**Published:** 2022-09-08

**Authors:** Wenhua Zhang, Shuwen Zhang, Chenlu Dong, Shuaijie Guo, Weiyu Jia, Yijia Jiang, Churan Wang, Mingxue Zhou, Yanbing Gong

**Affiliations:** ^1^ Dongzhimen Hospital, Beijing University of Chinese Medicine, Beijing, China; ^2^ Beijing Hospital of Traditional Chinese Medicine, Capital Medical University, Beijing, China; ^3^ Cardiovascular Disease Research Department, Beijing Institute of Chinese Medicine, Beijing, China

**Keywords:** bibliometric analysis, diabetes mellitus, diabetes complications, N6-methyladenosine (m 6 A), RNA methylation

## Abstract

**Background:**

RNA methylation has emerged as an active research field in diabetes mellitus (DM) and its complications, while few bibliometric analyses have been performed. We aimed to visualize the hotspots and trends using bibliometric analysis to provide a comprehensive and objective overview of the current search state in this field.

**Methods:**

The articles and reviews regarding RNA methylation in DM and its complications were from the Web of Science Core Collection. A retrospective bibliometric analysis and science mapping was performed using the CiteSpace software to plot the knowledge maps and predict the hotspots and trends.

**Results:**

Three hundred seventy-five qualified records were retrieved. The annual publications gradually increased over the past 20 years. These publications mainly came from 66 countries led by Canada and 423 institutions. Leiter and Sievenpiper were the most productive authors, and Jenkins ranked first in the cited authors. *Diabetes Care* was the most co-cited journal. The most common keywords were “Type 2 diabetes”, “cardiovascular disease”, “diabetes mellitus”, and “n 6 methyladenosine”. The extracted keywords mainly clustered in “beta-cell function”, “type 2 diabetes”, “diabetic nephropathy”, “aging”, and “n6-methyladenosine”. N6-methyladenosine (m^6^A) in DM and its complications were the developing areas of study.

**Conclusion:**

Studies on RNA methylation, especially m^6^A modification, are the current hotspots and the future trends in type 2 diabetes (T2D) and diabetic nephropathy (DN), as well as a frontier field for other complications of DM. Strengthening future cooperation and exchange between countries and institutions is strongly advisable to promote research developments in this field.

## 1 Introduction

DM is a common complicated chronic glycolipid metabolic disorder syndrome characterized by insulin resistance (IR) and insulin deficit (1). DM causes damage to major organs, including kidneys, heart, eyes, nerves, and blood vessels, eventually leading to diabetes complications that seriously threaten the health of individuals (1). According to the 10^th^ edition of the International Diabetes Federation (IDF) Diabetes Atlas, DM has been one of the fast-growing health emergencies of the 21^st^ century and has ranked among the top causes of premature death (2). However, the pathogenesis of DM remains unclear, and it is urgently essential to keep up with the current hotspots to explore the pathogenesis of DM and its complications.

RNA methylation may post-transcriptionally regulate RNA stability, localization, transport, splicing, and translation (3). More than 170 RNA modifications have been identified (4), of which m^6^A is the most abundant epigenetic modification in eukaryotic RNA. The abundance and effects of m^6^A on RNA are dynamically regulated by the interplay of methyltransferase, demethylases, and binding proteins. The methyltransferases that perform the modification reaction consist of Methyltransferase Like 3 (METTL3), METTL14, Wilms Tumor 1 Associated Protein (WTAP), etc. Reverse-modified demethylases known as “erasers” include fat mass and obesity-associated protein (FTO), α-ketoglutarate-dependent dioxygenase alkB homolog 5 (ALKBH5), etc. RNA-binding proteins are known as “readers” mainly composed of YTH domain-containing family 1/2/3 (YTHDF1/2/3), and YTH domain-containing protein 1/2 (YTHDC1/2) (5). M^6^A-mediated post-transcriptional modification is essential in many biological processes such as cell differentiation, stresses, circadian rhythm, cycle regulation, and metabolism (6). Emerging evidence revealed that m^6^A was involved in many human diseases and provided a potential novel pathogenesis study for the prevention or treatment of various diseases (7), such as cancer, cardiovascular disease (CVD), progeroid syndromes, autoimmune diseases, and metabolic diseases (8–12).

The complicated pathogenesis, as well as the lasting multi-organ damage of DM, made RNA methylation an increasingly interesting topic with a measurable increase in publications. Several studies identified the abnormal m^6^A levels and key methylesterase of m^6^A. For example, several studies monitored the decreased m^6^A in T2D and β-cells (13, 14). Correspondingly, changes in the levels of methylesterase were also monitored, such as METTL3, METTL14, WTAP, FTO, ALKBH5, and YTHDF1 in T2D and its complications (13–15). The abnormal change in m^6^A and key methylesterase affected cell proliferation, differentiation, apoptosis, and autophagy and led to abnormalities in cell structure and function that participated in the initiation and progression of DM and its complications (16). The current evidence from these studies reported that the upregulated or downregulated levels of m^6^A were connected with β-cells, renal tubular cells, IR, inflammatory, oxidative stress, lipid levels, obesity, and other unknown mechanisms, which aggravated hyperglycemia and its further damage to other organs (17). Therefore, a systematic review and summary of the topical and major findings of RNA methylation in DM and its complications based on the current literature is urgent and necessary at this stage.

Unlike general systematic reviews, bibliometrics is characterized by mathematical techniques to investigate publication and communication patterns in the distribution of information and to evaluate research trends qualitatively and quantitatively based on bibliographic databases and bibliometrics (18). It not only helps researchers to grasp the hotspots and trends of specific research fields but also helps to quantify the characteristics of the countries, journals, and authors of published articles (19). This analytical method has been widely used in various fields to develop guidelines, understand research hotspots, and evaluate research trends (20). Therefore, this review was conducted using bibliometrics analysis to identify essential evidence in RNA methylation for DM and its complications and to help scholars understand the intellectual backgrounds and the emerging research trends in this field.

## 2 Materials and methods

### 2.1 Data sources and search strategy

The Web of Science Core Collection is the largest comprehensive academic information resource covering multiple disciplines (21). To ensure the quality and accessibility of data, all data were downloaded from the Web of Science Core Collection online database with the queries shown in **Supplementary Table 1**, with no language or region restrictions. The document types were articles or reviews with dates ranging from 1 January 2002 to 28 May 2022.

### 2.2 Data collection and analysis

**Figure 1:**
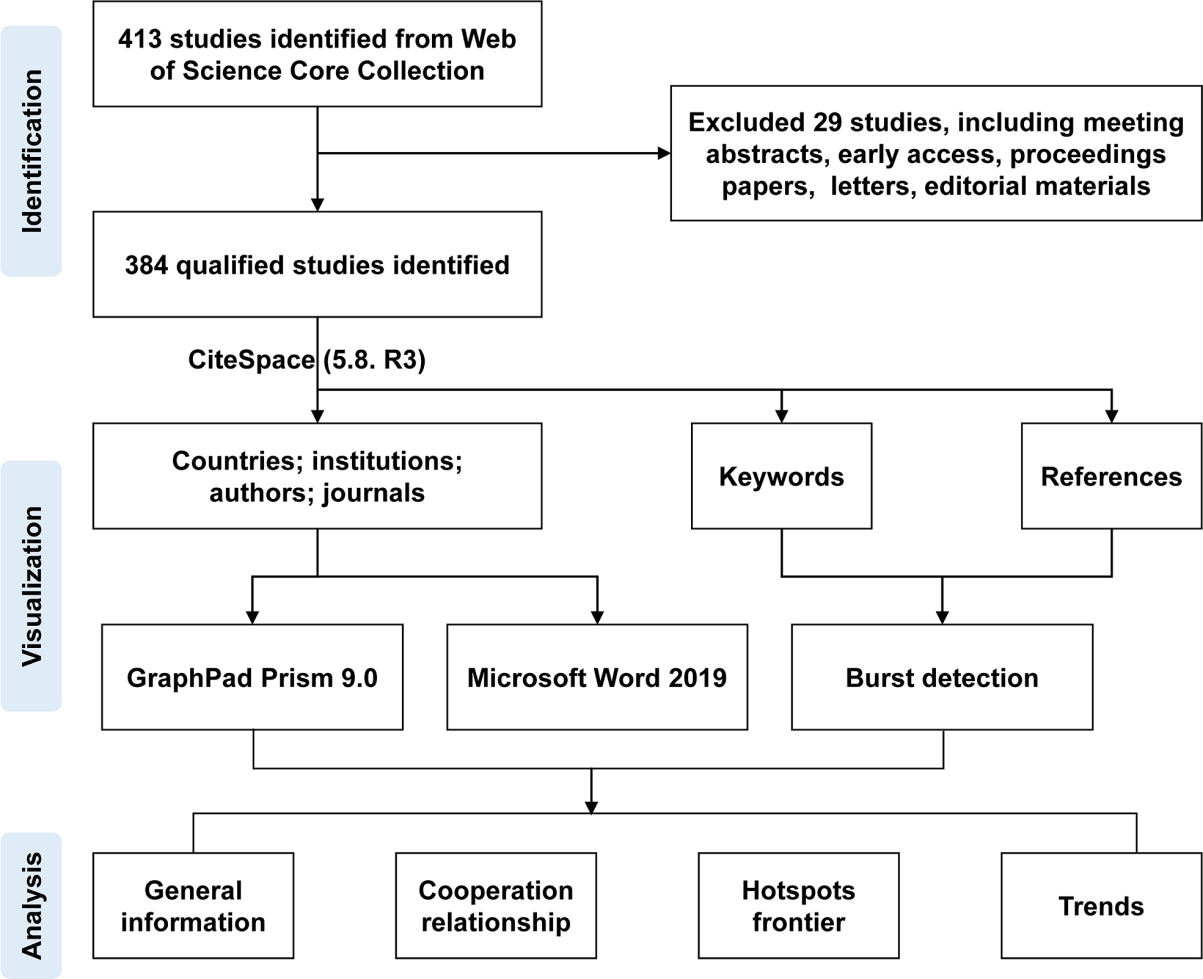
Flowchart of the review.

Two authors collected the documents from the Web of Science Core Collection according to the set retrieval mode, exported them to a plain text file with full records and cited references, and stored them in download_txt format. Any disagreements were resolved by consensus. All valid data were converted to Microsoft Excel 2019, CiteSpace (5.8. R 3), and GraphPad Prism 9.0, and the review flowchart is shown in **Figure 1**.

CiteSpace is a Java application for visualizing and analyzing scientific literature trends and patterns (22). It not only helps conduct burst detection, intermediate centrality, and heterogeneous networks and identify and track critical paths and milestone studies during professional development but also helps detect emerging trends and transient patterns at the research forefront (21). Therefore, this review used the CiteSpace software to conduct a bibliometric analysis of the publications of RNA methylation in DM and its complications, including countries, institutions, authors and cited authors, journals, references and keywords, the bursts of references and keywords, an overlay of journals, and a timeline viewer of keyword clusters.

Microsoft Word 2019 was used to conduct the descriptive statistics on ranks, frequency, attributes, centrality, etc. GraphPad Prism 9.0 was used to analyze and plot the annual publication output. The web tool MapInSeconds (mapinseconds.com) of *Eugene Chen, Darkhorse Analytics* was used to draw the geographical distribution of article publications.

## 3 Results and analysis

### 3.1 The trend of publication outputs

**Figure 2:**
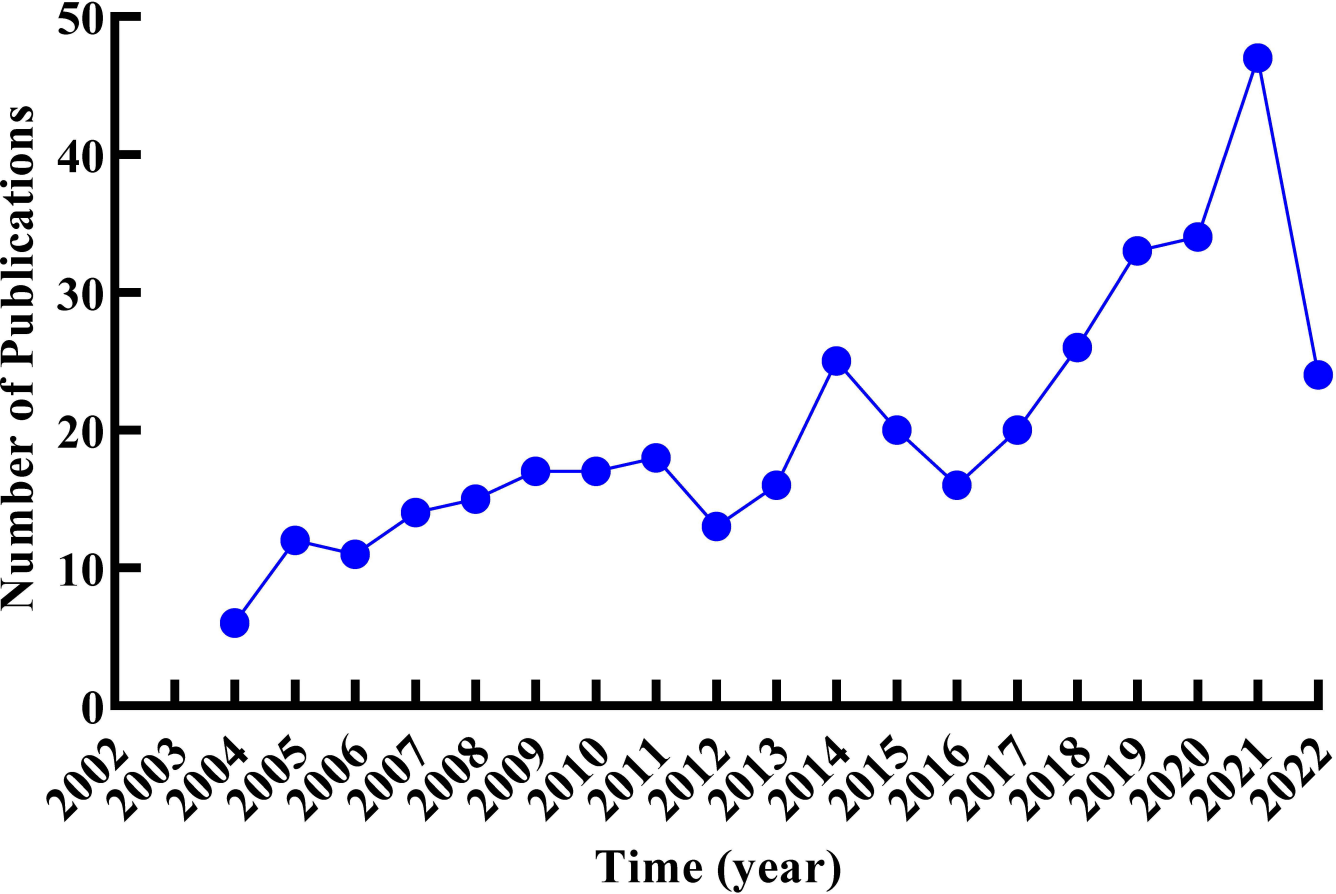
Trends of the annual publications related to RNA methylation in DM and its complications.

A total of 384 documents were collected from the Web of Science Core Collection based on the predefined queries. CiteScape identified no duplications, and 373 qualified records were included in this review. As shown in **Figure 2**, the research trend was divided into three stages. The first stage with 110 literatures was from 2004 to 2011, when the outputs of literature gradually rose from 6 to 18. There was a slight fluctuation in the second phase, with 13 articles in 2012, 25 articles in 2014, and a drop to 16 articles in 2016. From 2017 was the third stage; annual outputs climbed gradually steadily without any decline, as of 28 May 2022, out of 184 articles. In 2022, only 5 months of publication were counted; it cannot represent the whole annual outputs, but the overall trend in outputs was increasing on RNA methylation in DM and its complications, which suggests that RNA methylation may be a promising direction in this field.

### 3.2 Analysis of countries/regions distribution

**Figure 3:**
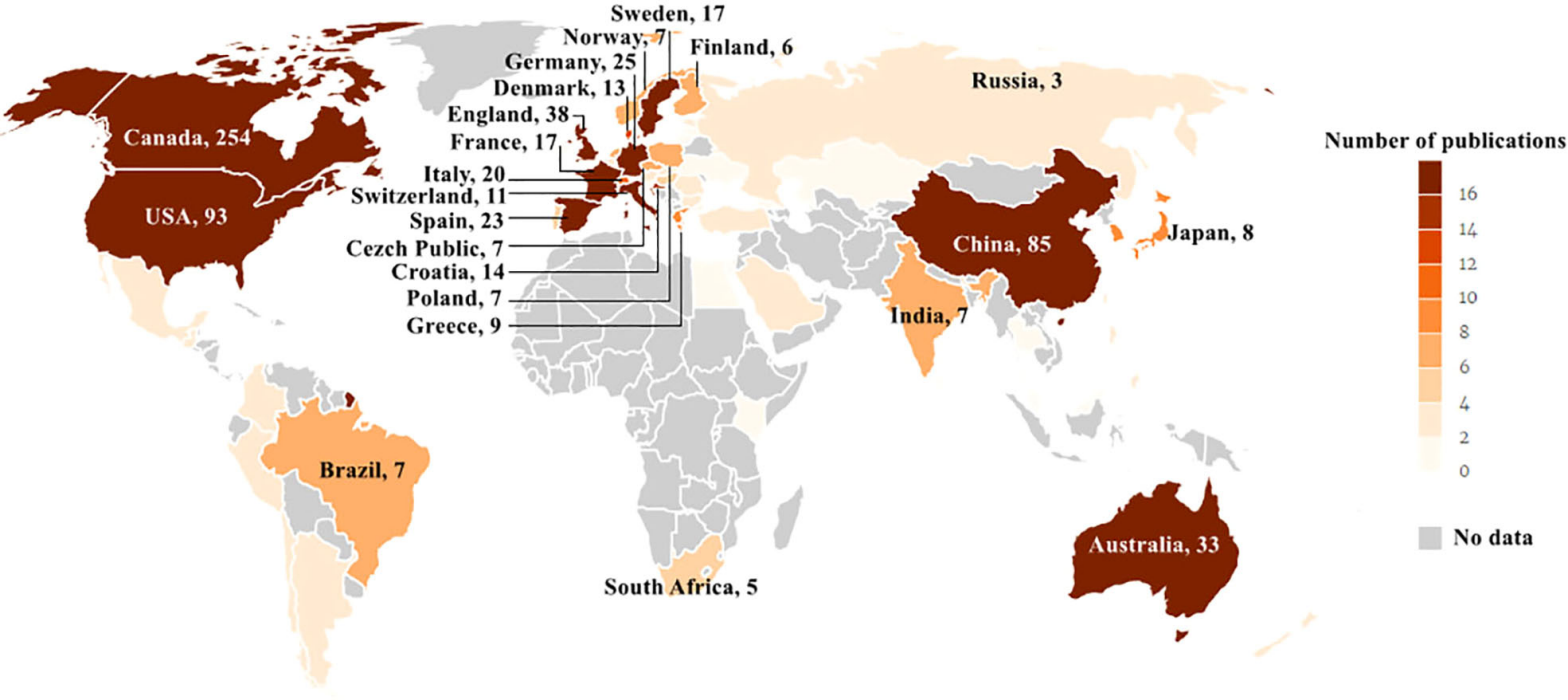
Geographical distribution of article publications related to RNA methylation in DM and its complications. The label represents the country and the outputs of the country; the depth of color matched with the number of publications.

**Figure 4:**
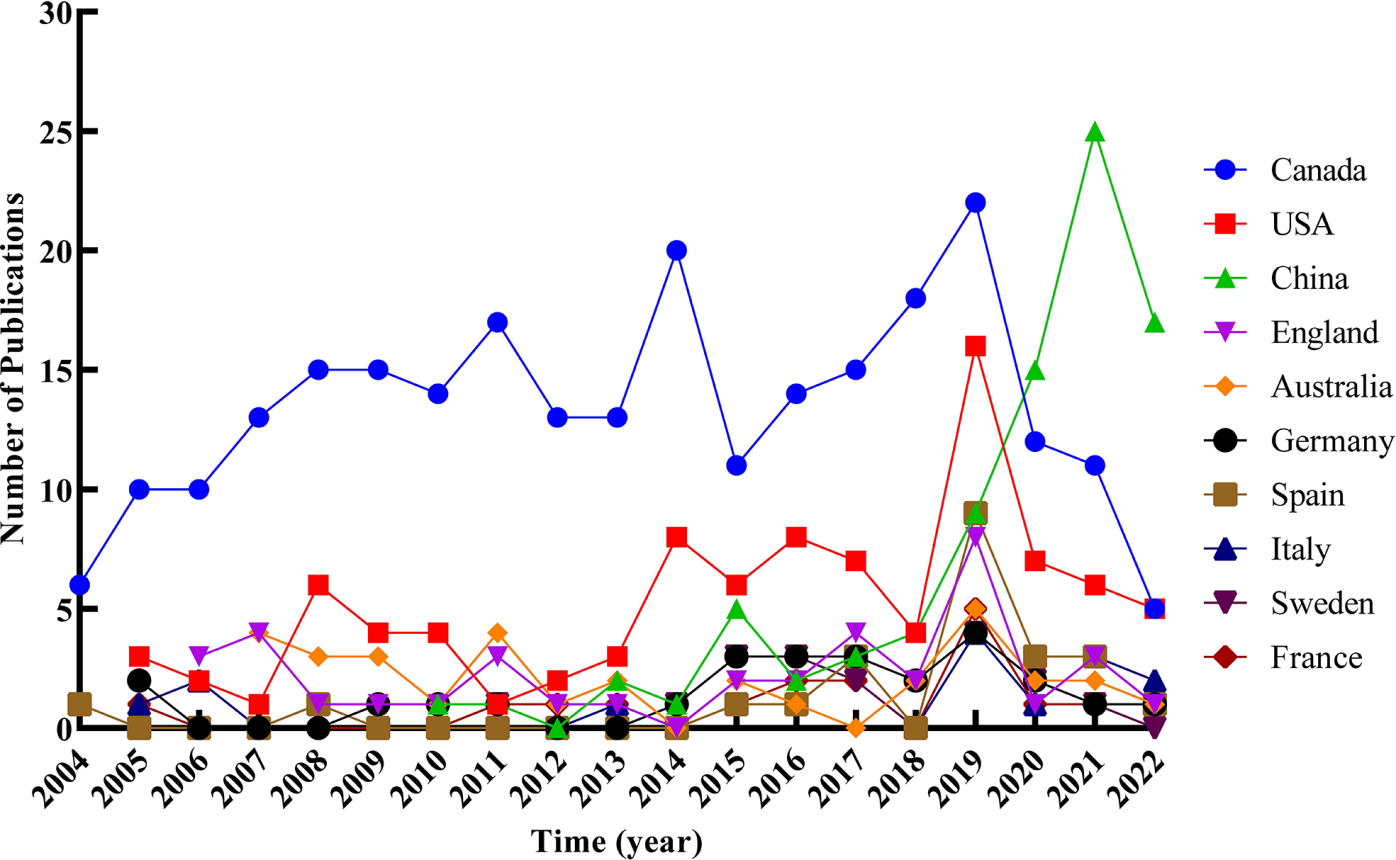
Trends of annual publications of the top 10 countries/regions related to RNA methylation in DM and its complications.

Three hundred seventy-three records were published in 66 countries/regions in the five continents. The deeper color in **Figure 3** indicates that the publications were higher in this country, while the gray indicates no articles published. Canada ranked first with the highest number of 245 articles in the field. The USA with 93 articles ranked second, and China with 85 articles ranked third. Both were far ahead of other countries. Annual outputs of the top 10 countries/regions are shown in **Figure 4**; Canada posted significantly more annual outputs than any other country until 2019, after which Canada and USA decreased, while China overtook other countries to rank first in annual productions.

**Figure 5:**
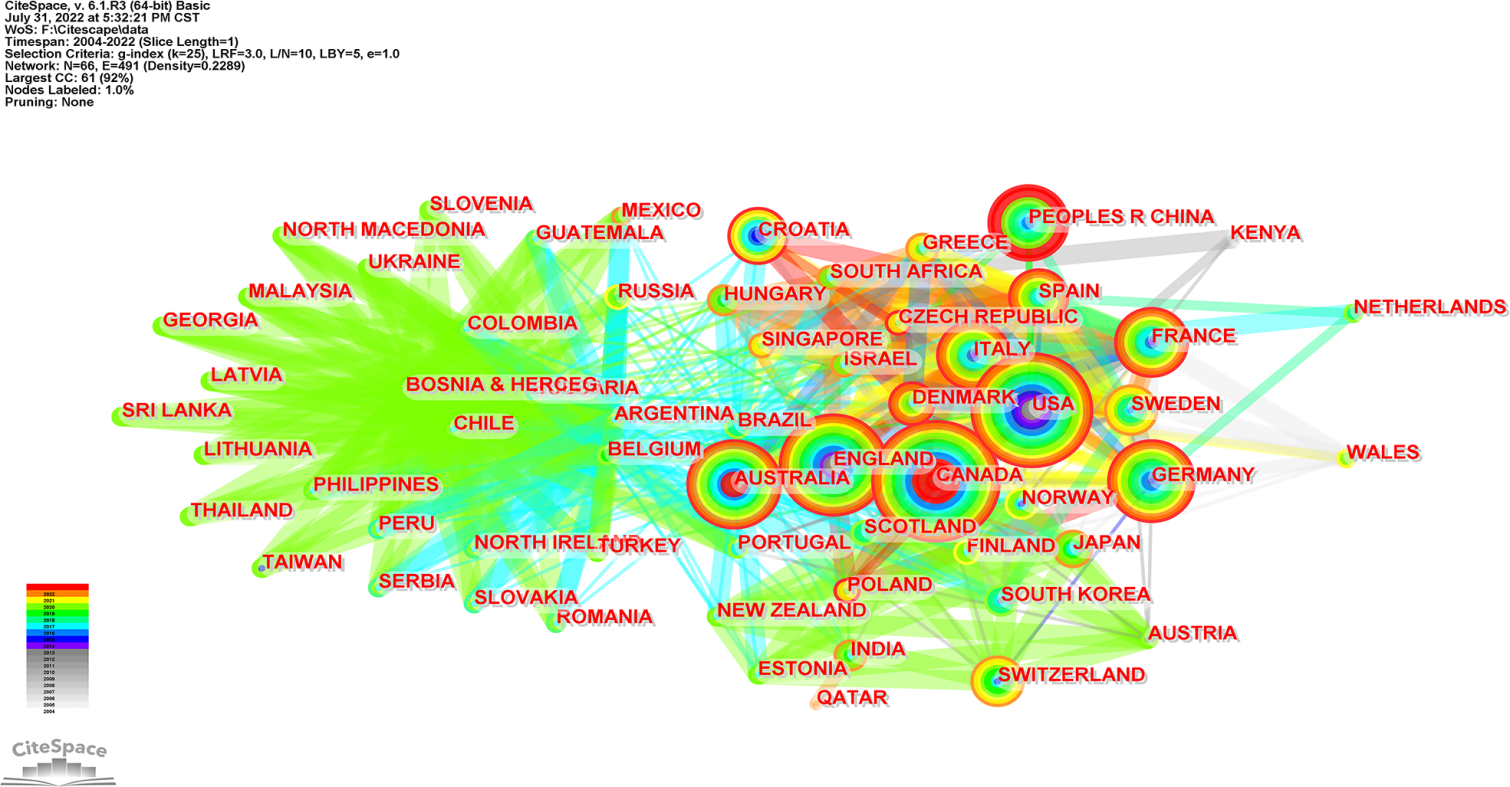
Visualization map of countries/regions collaboration analysis related to RNA methylation in DM and its complications. Each node with colorful annual rings represents a country; the color bandwidth corresponds to the outputs of the year; the wider the ribbon, the higher the number of outputs posted and the corresponding node increases. The purple color around the nodes represents high centrality. Link between the two nodes represents the cooperation relationship, and the link color represents the initial cooperation year.

The collaboration of countries/regions is visualized in **Figure 5**. Each node with colorful annual rings represents a country, and the size of the node matches the outputs in this country; the color bandwidth corresponds to the outputs for this year, and the wider the ribbon, the higher the number of outputs posted. The results in **Figure 5** matched the geographical distribution in **Figure 3**. The purple color around the nodes represents high centrality. Belgium, Argentina, and Australia were circled in purple, which indicates they have a strong bridge role in this field. The link density between two nodes matches the cooperation strength, and the link color represents the initial cooperation year. Although relatively few articles were issued, Belgium and Argentina had the most cooperative relations with other countries. Among the top countries, Australia had a higher centrality and more cooperation such as Canada, the USA, China, England, Germany, etc.

### 3.3 Analysis of institutions distribution

A total of 375 qualified articles were published by 423 institutions. As shown in **Supplementary Table 2**, Univ Toronto (205) and St Michaels Hosp (154) were marked leaders in the publications, followed by McMaster Univ and Univ Saskatchewan. Of the top 10 institutions, seven were in Canada and two were in the USA.

**Figure 6:**
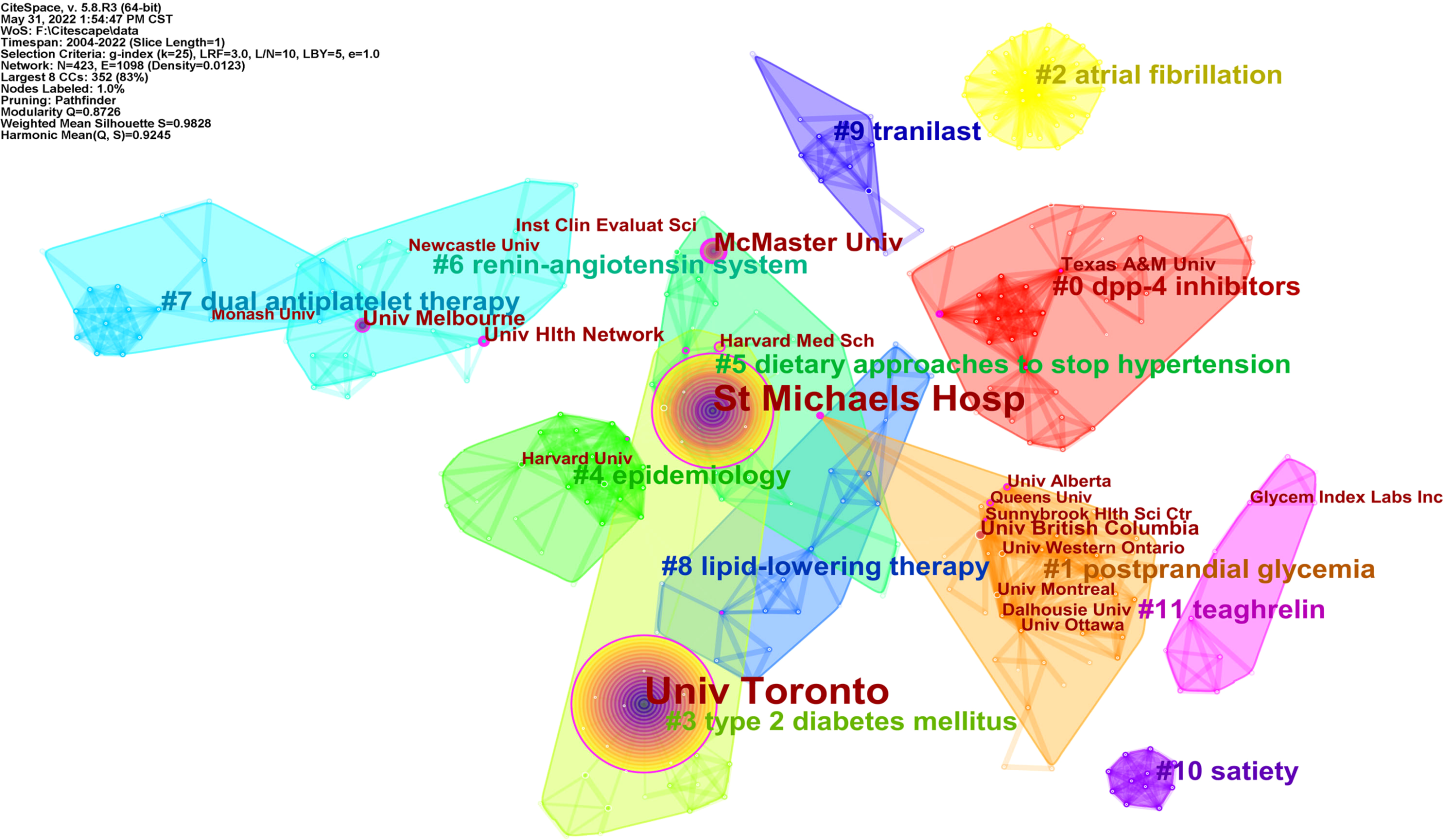
Visualization map of institution collaboration analysis related to RNA methylation in DM and its complications. Each node with colorful annual rings represents an institution. Institutions with partnerships make up the different color groups. The text with # represents the keyword clusters of the institutions.

Institutional co-occurrence analysis reflects the collaboration relationship. The map with a network density of 0.0123 comprised 423 nodes and 1368 links. As shown in **Figure 6**, the Univ Toronto and St Michaels Hosp circled in purple were the two largest nodes, with the highest literature and the strongest centrality, which implied an essential role in this field. The thickness of the line between nodes indicates institution collaboration. Most institutions collaborated, for example, McMaster Univ, St Michaels Hosp, Heart and Stroke Fdn Ontario, Tech Univ Dresden, and so on.

To explore the research themes among institutions, log-likelihood tests (LLR) were used to cluster the keywords of the articles published by the institutions by CiteSpace, and the top 10 clusters with different colored areas are shown in **Figure 6**. “Cluster #0 dpp 4-inhibitors” is the largest cluster, followed by “cluster #1 postprandial glycemia”. The top two institutions are clustered in “#3 type 2 diabetes mellitus”, which was the focus of the two institutional studies.

### 3.4 Analysis of authors and cited authors

These publications involved 511 authors, with an average of 1.37 authors per article (**Supplementary Table 3**). The first eight authors were all from Canada. Leiter was the highest producer with 44 articles; the rest of the authors in this field had more than 10 articles. Jenkins had received 55 citations and was the first cited author, followed by Wolever, Wang, and Sievenpiper.

**Figure 7:**
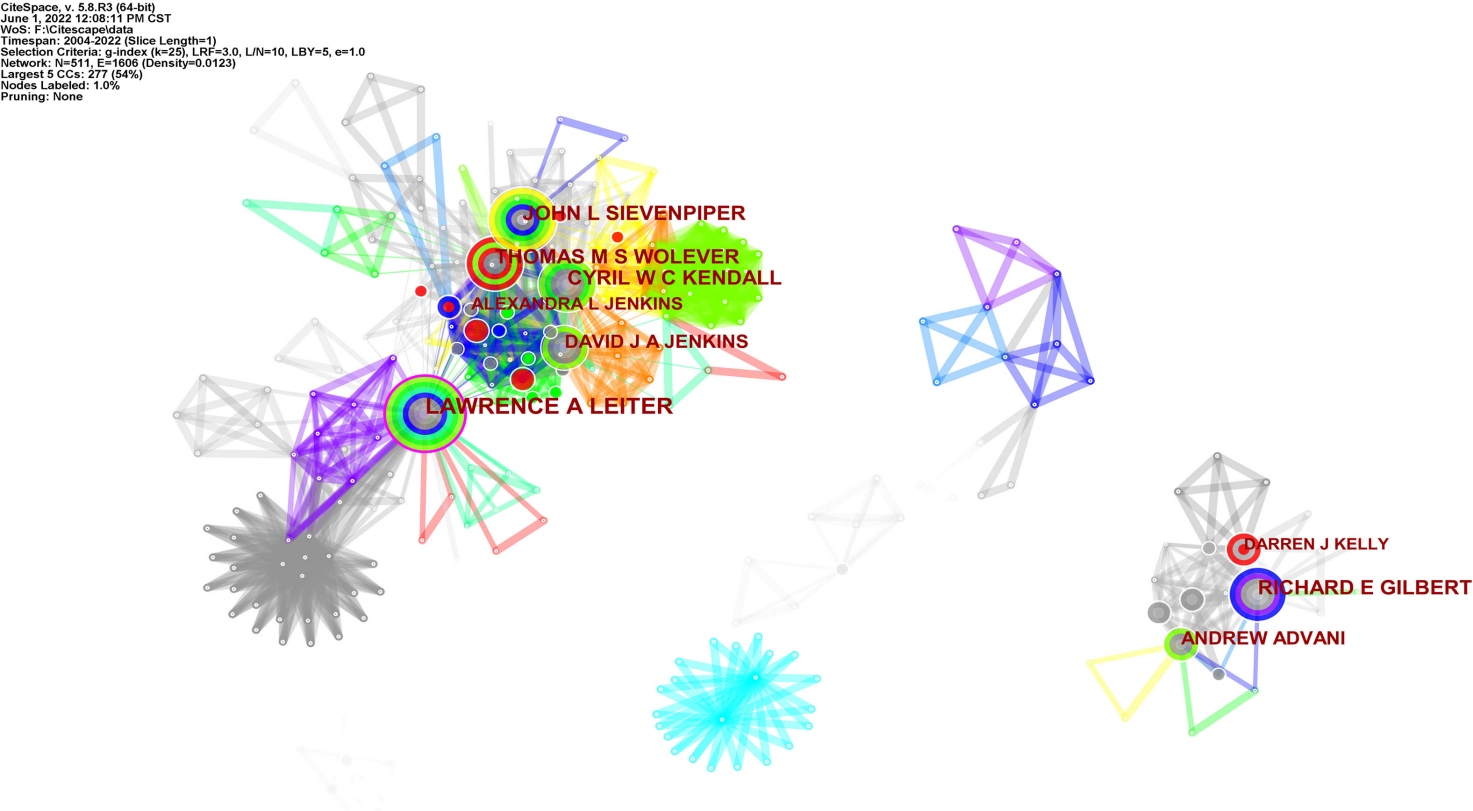
Visualization map of the top five author collaboration related to RNA methylation in DM and its complications. Each node with colorful annual rings represents an author. The size of the nodes matches the publications of the author. The nodes circled in purple represent greater centrality; the nodes circled in red represent higher burst. The separate areas made up of nodes and links represent the author collaborative relationships.

When author collaborations were analyzed, **Figure 7** visualizes the top five author collaboration groups. Leiter, Sievenpiper, Kendall, and others worked together for years and formed the largest collaborative team, of which Leiter is circled in purple for greater centrality and Wolever with conspicuous red for higher burst. Gilbert, Advani, Kelly, and other authors made up the second collaboration team, of which Kelly is circled red for having publication burst growth in a short time.

### 3.5 Analysis of cited journals

Co-citation analysis of the journal in the references reveals the authoritative and influential journal in this field. Among the 517 cited journals, eight were cited over 100 times (**Table 1**). *Diabetes Care* was cited most, while *New England Journal of Medicine* and *Diabetes* followed second. Of the top 10 cited journals, nine were distributed in the Q1 region, with only *PLoS One* belonging to Q2. Among the top 10 journals, the *New England Journal of Medicine* had the highest impact factor (IF) of 91.253, and *Lancet* ranked second with an IF of 79.323.

**Table 1:** The top 10 co-cited journals related to RNA methylation in DM and its complications.

No.	Co-cited Journal	Citation	Centrality	IF (2020)	JCR
1	*Diabetes Care*	202	0.01	19.112	Q1
2	*New England Journal of Medicine*	168	0.01	91.253	Q1
3	*Diabetes*	154	0.07	9.461	Q1
4	*Lancet*	152	0.03	79.323	Q1
5	*JAMA—Journal of the American Medical Association*	141	0.05	56.274	Q1
6	*Diabetologia*	138	0.01	10.122	Q1
7	*Circulation*	130	0.02	29.69	Q1
8	*Nature*	118	0.07	49.962	Q1
9	*PLoS One*	99	0.03	3.24	Q2
10	*American Journal of Clinical Nutrition*	98	0.04	7.047	Q1

**Figure 8:**
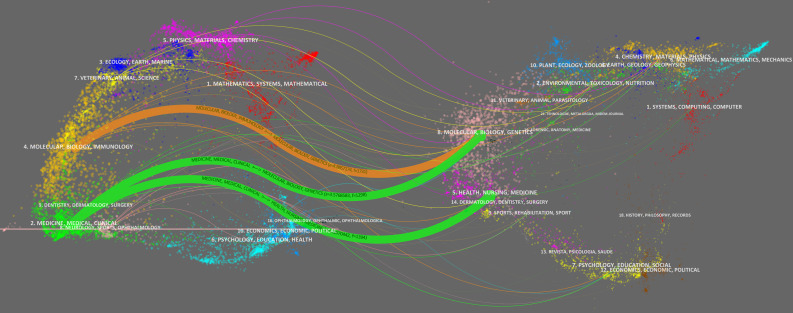
The dual-map overlay of journals on RNA methylation in DM and its complications. The left nodes represent the included literature; the right represent the references in the literature. The labels represent the discipline. The link represents the cited path.

The dual-map overlay of journals reflects the relationship between the source journals on the left and the target journals on the right, as well as the subjects involved in the journals. As shown in **Figure 8**, the colorful links represent the relationship between the two journals; there were three main wide citation paths, namely two green paths and one green path. The green paths indicated that the journals that involved molecular, biology, genetics and health, nursing, and medicine were always cited by medicine, medical, and clinical journals. The orange paths indicated that the journals that involved molecular, biology, and genetics were cited by molecular, biology, and immunology.

### 3.6 Analysis of co-cited references and reference burst

When two or more references are cited by several articles, the two references are considered to be a co-citation relationship. Among the 695 cited references, the top eight most frequently co-cited references are shown in **Table 2**. The reference published by Yang (13) had the highest number of citations, which implied that the research related to this article may be a research hotspot, and five references were cited more than 10 times and were valuable literature in this field.

**Table 2:** Top eight co-cited references related to RNA methylation in DM and its complications.

No.	Author	Year	Reference	Citation	Centrality
1	Yang Y	2019	Glucose is involved in the dynamic regulation of m^6^A in patients with type 2 diabetes	16	0.02
2	Roundtree IA	2017	Dynamic RNA modifications in gene expression regulation	15	0
3	Huang HL	2018	Recognition of RNA N-6- methyladenosine by IGF2BP proteins enhances mRNA stability and translation	14	0.19
4	Bhattacharyya OK	2008	Management of cardiovascular disease in patients with diabetes: the 2008 Canadian Diabetes Association guidelines	14	0
5	De Jesus DF	2019	m^6^A mRNA methylation regulates human beta-cell biology in physiological states and in type 2 diabetes	13	0.01
6	Xie W	2019	METTL3 inhibits hepatic insulin sensitivity *via* N6-methyladenosine modification of Fasn mRNA and promoting fatty acid metabolism	9	0
7	Shi HL	2017	YTHDF3 facilitates translation and decay of N-6-methyladenosine-modified RNA	9	0
8	Lin SB	2016	The m^6^A Methyltransferase METTL3 promotes translation in human cancer cells	9	0

**Figure 9:**
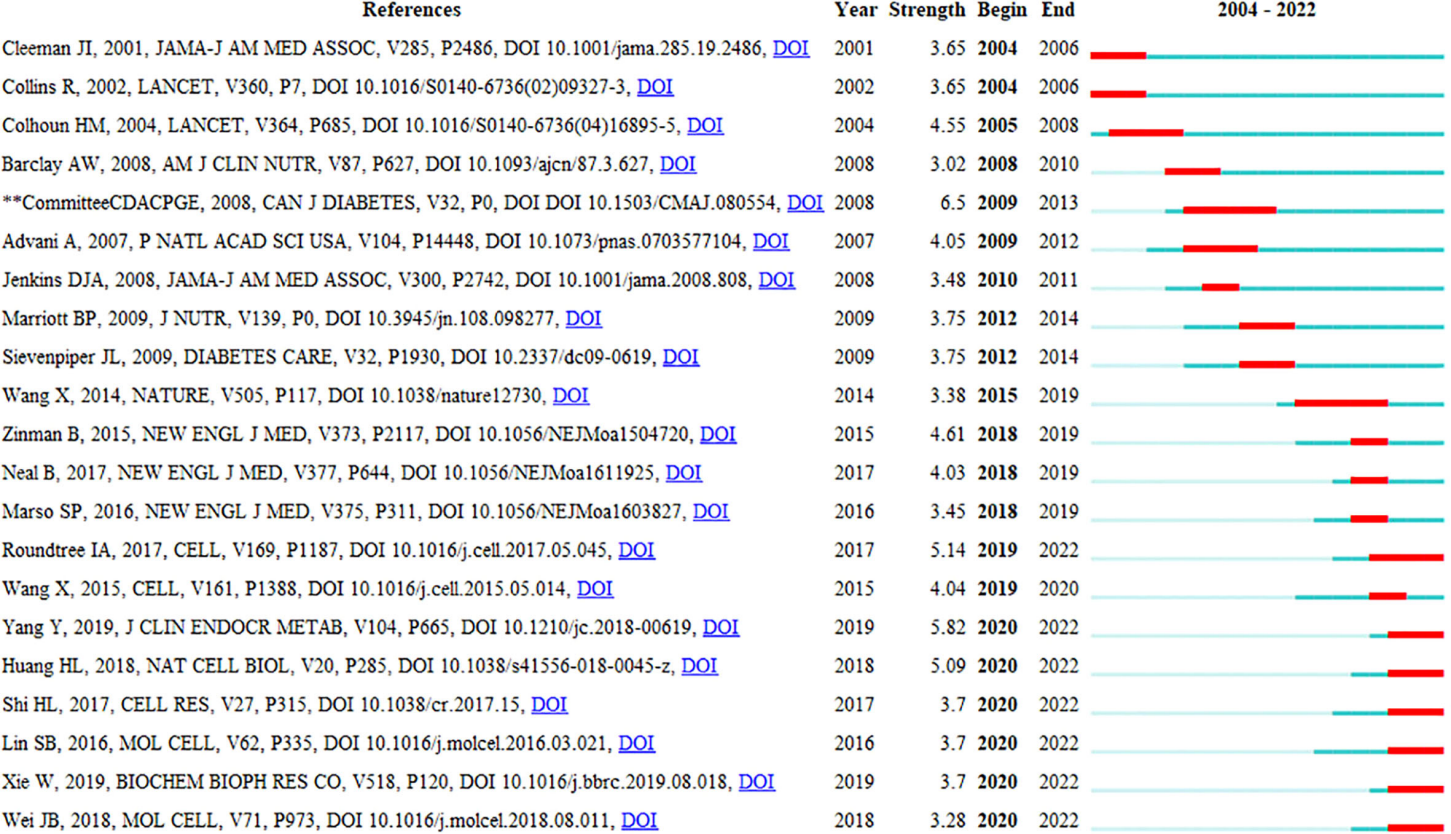
Top 21 references with the strongest citation bursts related to RNA methylation in DM and its complications.

Reference burst detection helps select the bulged references in a short time from the numerous references and find the most influential cited articles, thus clearly discovering the research frontiers and trends. **Figure 9** displays the reference citation burst detection. The reference written by Bhattacharyya (23) had the highest centrality and burst strength and was the bridge between the two related studies, representing the current research hotspot and a turning point. The references published by Committee CDACPGE (2008) and Wang (2014) had the longest burst duration, which suggested a longer active duration of the research. Judged from the last 3 years, the references published by Yang (13), Roundtree (3), and Huang (2018) had a stronger burst, which had become the latest research frontier so far and may continue in the future.

### 3.7 Analysis of hotspots and frontiers

Keywords represent the research hotspots. We counted the keywords in the literature by CiteSpace and merged the top similar keywords and obtained the top 20 high-frequency keywords in **Table 3**; “type 2 diabetes”, “cardiovascular disease”, “diabetes mellitus”, “n 6 methyladenosine”, and “expression” had higher frequency. It indicated that they were the popular topics on RNA methylation in DM and its complications.

**Table 3:** The top 20 keywords related to RNA methylation in DM and its complications.

No.	Keywords	Count	Centrality
1	type 2 diabetes	51	0.21
2	cardiovascular disease	48	0.14
3	diabetes mellitus	38	0.18
4	n 6 methyladenosine	35	0.04
5	expression	33	0.14
6	coronary heart disease	33	0.11
7	disease	29	0.23
8	risk	27	0.3
9	glucose	26	0.13
10	diabetic nephropathy	25	0.11
11	blood pressure	24	0.08
12	insulin resistance	23	0.14
13	mortality	17	0.08
14	messenger RNA	16	0.08
15	association	16	0.07
16	risk factor	16	0.05
17	metaanalysis	15	0.08
18	insulin	14	0.1
19	gene expression	14	0.02
20	RNA	12	0.01

**Figure 10:**
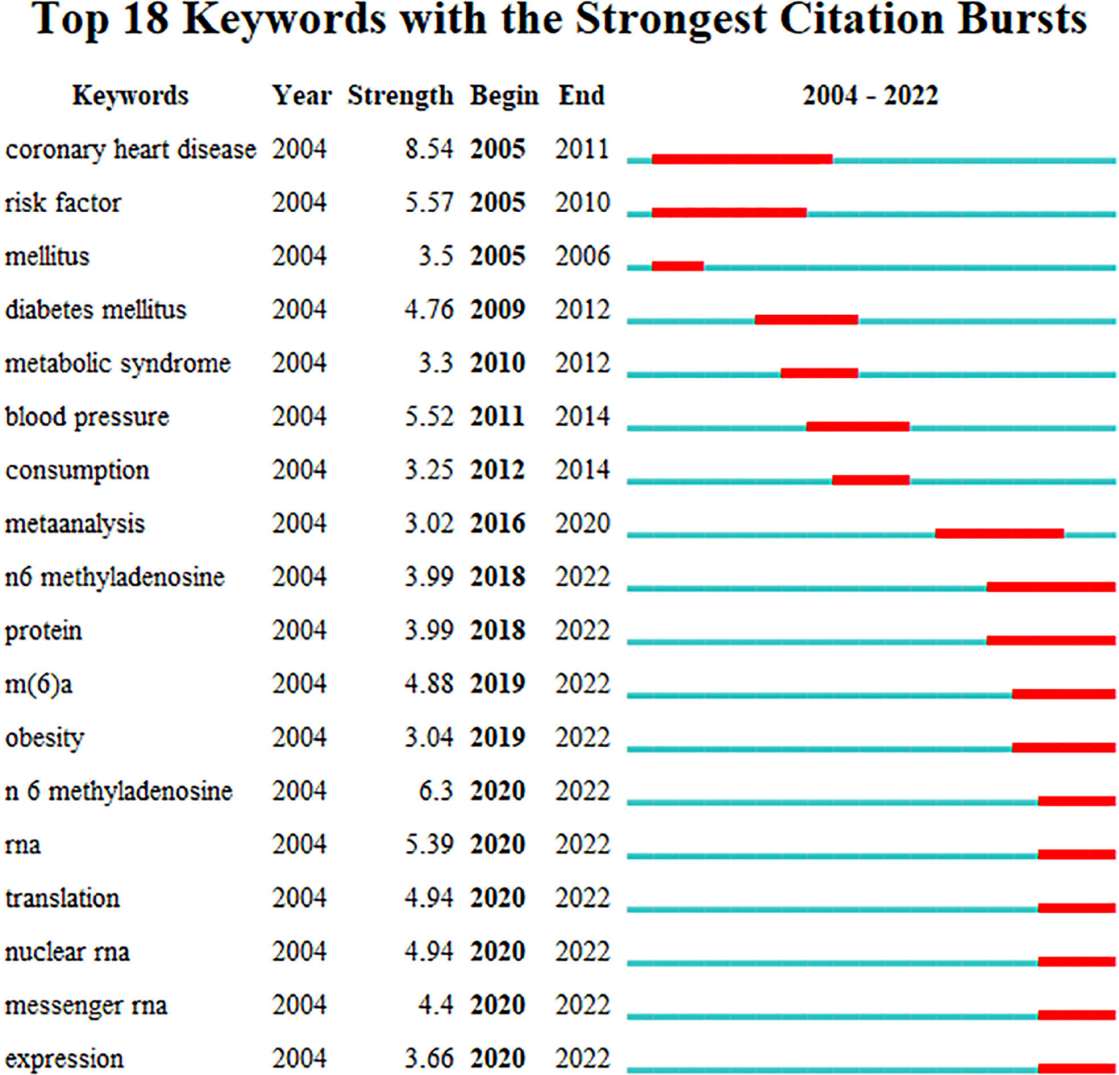
Keyword bursts related to RNA methylation in DM and its complications.

Keywords bursts were detected to review and predict the phased hotpots and their evolutionary trends of RNA methylation in DM and its complications. As shown in **Figure 10**, “coronary heart disease”, “risk factors”, and “mellitus” with stronger bursts of strength emerged earlier and were the topics of early attention. The keywords “mellitus” and “diabetes mellitus”, as synonymous terms, had the longest, 7 years of duration burst, followed by “coronary heart disease”. Since 2018, the keywords related to RNA methylation have begun to appear and have continued until now. “N 6 methyladenosine” with the strongest burst suggested that it was the hotspot and maybe a turning point with prospective research implications.

**Figure 11:**
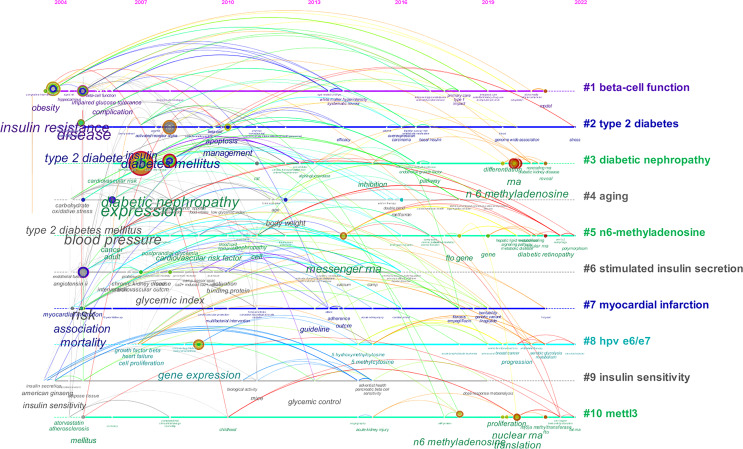
CiteSpace visualization timeline view of keywords clustering analysis related to RNA methylation in DM and its complications. The different colored horizontal lines with the label on the right represent the clusters formed by the keywords, the nodes on the horizontal lines represent the keywords, and the position of the nodes on the horizontal lines represents the year in which the literature containing the keywords first appeared, thus forming the timeline of the keywords cluster evolution.

Keywords clusters were carried out and presented in a timeline view to observe the basic knowledge structure and the evolution over time of RNA methylation in DM and its complications. A total of 30 clusters were obtained through CiteSpace. The 1–10 clusters were presented in **Figure 11**. The nodes are chronicled on the horizontal line, which evolved the historical outcomes of the cluster. The clusters’ names are listed in order on the right. Cluster “#1 beta-cell function” was the largest cluster and “#2 type 2 diabetes” was the second cluster; “#10 mettl3” was the last one. “#1 beta-cell function” appeared earliest and “#6 stimulated insulin secretion” appeared latest. Cluster “type 2 diabetes”, “n6-methyladenosine”, “hpv e6/e7”, and “mettl3” related studies were available in 2022, while “aging”, “stimulated insulin secretion”, and “insulin sensitivity” gradually decreased or even disappeared, suggesting a decreased trend in this field. The colored annual node on the horizontal line represents keywords; the position on the line indicates the time of the first literature, and the width of the annual color band matches the amount of literature in this year. It was observed that the nodes of “expression”, “diabetic nephropathy”, “insulin resistance”, “type 2 diabetes”, and “diabetes mellitus” were larger, which indicated that there were more published articles on related studies. “Risk”, “expression”, and “type 2 diabetes” circled in purple had a higher centrality and implied a significant link role in this field. The number of nodes in cluster “diabetic nephropathy”, “n6-methyladenosine”, and “mettl3” grew in recent years and may be the frontier on RNA methylation in DM and its complications in the future.

## 4 Discussion

### 4.1 General information

The output of publications at a particular stage reflects the trends in the research themes. The trends in annual publications showed an overall upward in the number of publications. The period from 2004 to 2011 was a nascent period with few articles published and a waved rise in publications. From 2012 to 2016, the research entered the exploration period. In the last 5 years, the research entered a critical period along with the rapid growth of the literature. It was evident that the research related to RNA methylation in DM and its complication was a hot topic in recent years and had a positive future trend.

Visualization results are based on countries, institutions, and authors in the literature. Canada was the leading country with the highest publications and almost all of the top 10 institutions and authors were from Canada in this field. China has also gradually invested in this field with increasing publications in the last 3 years. However, Canada and China do not have a higher centrality, which implied that the academic cohesion was still insufficient and the bridge role was insignificant. As for the collaboration relationship, there was a collaborative network between countries, but the relationship was relatively loose, and most cooperative institutions and researchers were limited to internal connections, with less international cooperation. This situation may not be conducive to complementary strengths, thus preventing research development. Therefore, it is strongly advisable to import specialized talents or send our countries or institutional personnel to advanced institutions or renowned scholars for targeted training, such as Univ Toronto and St Michaels Hosp. It is necessary to promote international academic exchanges and accelerate research progress in the same field.

To some extent, the frequency of the co-citation reflects the intrinsic scientific value and research backgrounds. The frequency of the co-citation reflects the influence of the author in this field and the centrality reflects the connection with the other authors. Jenkins was the most co-cited author and had higher centrality, indicating a high level of influence in this field and a stronger connection to other authors. Wolever had the higher burst, which indicated that his published reports received a high level of interest over time. The *New England Journal of Medicine*, *Lancet*, and *Nature* were highly specialized journals and influenced the academic directions and the foundations of the field. The dual-map overlay reflects the major studies on RNA methylation in DM and its complications involving various disciplines, including molecular, biology, genetics, etc. Concerning the co-cited references, the most cited article by Yang (13) had a valuable contribution revealing alterations of m^6^A and methyltransferase in T2D (13). The article by Roundtree in 2008 had the highest centrality, reflected the relationship between CVD and DM, and reviewed recommendations for risk factor management in CVD (23).

### 4.2 Hotspots and frontiers

Keywords articulate the subject of the documents and the timeline viewer reveals the formation process of the cluster. Combined with some similar keywords, the keywords and their clustering showed that DM, T2D, DN, and insulin appeared frequently and had four clusters associated with them. T2D, which accounts for 90% of DM (24), also had a long duration in the timeline map and was the key point of the study. In the complications of DM, DN had the highest frequency and was clustered in #3; DN, as one of the common microvascular complications of DM, is the leading cause of chronic kidney disease (CKD) and end-stage renal disease (ESRD) (25). In addition, “risk factor” and “cardiovascular disease” appeared in the top 20 keywords. Several studies suggested that either hyperglycemia or hypoglycemia was associated with an increased risk of cardiovascular events and mortality, and CVD was the main cause of mortality among DM patients (23, 26). Thus, DM was the main axis of research in terms of frequency or importance. m^6^A, expression, RNA, and the clusters “#5 n6-methyladenosine” and “#10 mettl 3” were all involved in the RNA methylation. M^6^A was the most studied RNA modification implicated in many fundamental RNA metabolisms such as translation, splicing, stability, and decay (3, 4, 27). METTL3 mediates m^6^A methylation of mRNA, which affects the stability of mRNA and its translation into protein (28). M^6^A contributed to the development of RNA epigenetics and became a frontier research area.

**Figure 12:**
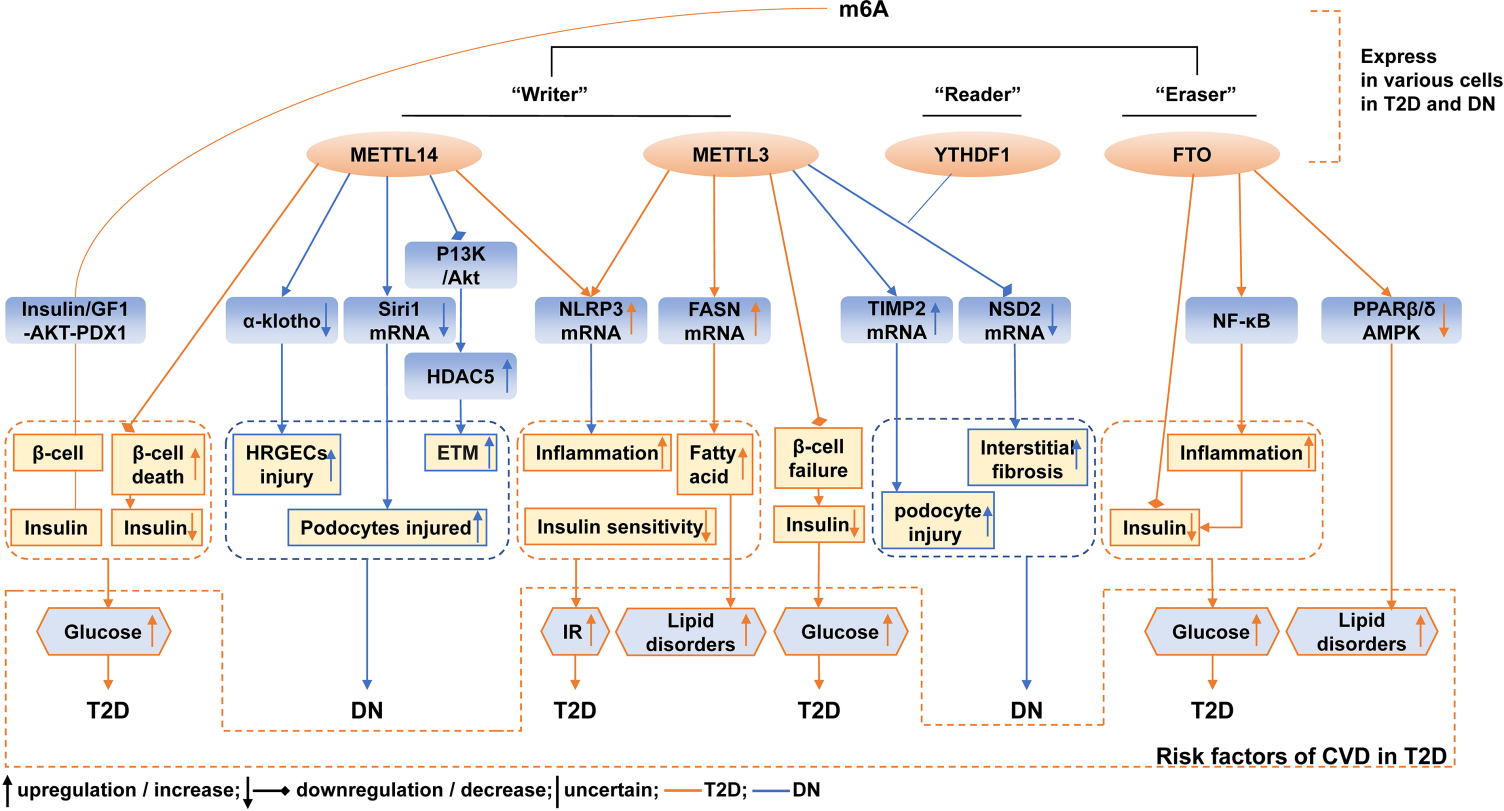
Schematic illustration of current mechanism of m^6^A and methylesterase in T2D and DN, and CVD risk factors associated with m^6^A and methylesterase in T2D. The orange lines represent mechanisms associated with T2D, and the blue lines represent mechanisms associated with DN.

Burst detection reveals the hotspots, frontiers, and trends. Co-cited references reflect the backgrounds and the baselines. Combining those keywords and keywords clusters, we screened the references related to m^6^A in T2D and DN and summarized the current research hotspots and trends in the following aspects; a brief diagram of the current status of research on m^6^A and methylesterases in T2D and DN and risk factors for CVD is shown in **Figure 12**.

#### 4.2.1 Change of m^6^A and methylesterase in T2D and DN

Currently, there are guidelines for diagnosing T2D and DN, but specific markers for its pathogenesis are still being explored. Emerging evidence confirmed the change of m^6^A in T2D and DN. The levels of m^6^A were reduced in the peripheral blood of T2D patients compared with healthy controls. The levels of m^6^A were negatively correlated with fasting blood glucose. The upregulated mRNA expression of FTO may be responsible for the reduction of m^6^A and was associated with the risk of T2D (14). Another study also reported the decreased level of m^6^A in white blood cells of patients with T2D compared with healthy individuals. The study demonstrated that the mRNA expression of the demethylase FTO was upregulated in white blood cells, and high glucose promoted FTO expression, which further induced m^6^A to decrease in T2D (13). Additionally, the levels of other m^6^A methylesterase were also varied in T2D. The mRNA levels of METTL3, METTL14, and WTAP were increased in the white blood cells of T2D patients compared with healthy individuals; the high-glucose stimulation promoted the abundance of FTO protein in both patients with T2D and HepG2 cells (13). In the islets, m^6^A and m^6^A methylesterases were also altered. FTO mRNA expression was lower in T2D islets than in non-diabetic islets (29). METTL3, METTL14, ALKBH5, and YTHDF1 were decreased in the β-cells of T2D patients than in non-diabetic individuals. METTL3 and METTL14 protein levels were also decreased in the whole islets from T2D patients (15). Therefore, alteration of m^6^A contents may be a specific biomarker for predicting the risk of T2D and its complications. However, these results should be repeated and validated in a larger population and different experiments. Furthermore, more studies are needed to identify the levels and corresponding causes of m^6^A and m^6^A methylesterase in T2D and to find their correlation with cellular function. This will help to facilitate the elucidation of the biological significance of m^6^A and methylesterase changes in T2D.

The m^6^A and m^6^A methylesterase levels were also detected in DN. Compared with db/m mice, in the renal tissue of streptozotocin-induced diabetic mice and db/db mice, m^6^A modification levels and METTL3 levels were significantly increased (30). In high-glucose-treated mouse mesangial cell lines, the m^6^A level was reduced, and the serum level of METTL3 was reduced in patients with DN compared with healthy individuals (31). In HK2, the decreased mRNA expressions of FTO, METTL3, and METTL14 were reported (32). The M^6^A level and expression of WTAP were increased in renal tubules but not the glomerulus from patients with DN and HK2 (33). M^6^A, METTL3, METTL14, and WTAP were significantly upregulated in the renal cortex of Adriamycin-treated mice than the corresponding controls, and METTL14 was also upregulated in the biopsy samples of patients with DN in comparison to healthy controls (34). In the high-glucose human renal glomerular endothelial cells (HRGECs), METTL14 was significantly increased compared with the normal-glucose HRGECs, and METTL14 was significantly increased in the kidney tissues of DN patients both at the mRNA and protein levels compared with the normal adjacent tissues of renal carcinoma patients (35). FTO expression was significantly reduced in the serum samples of DN patients compared with healthy volunteers (36). Together, these data revealed that the levels of m^6^A and m^6^A methylesterase varied in DN. However, due to the small number of current studies, more evidence is needed to identify the m^6^A and its regulators’ change in the kidney, which will facilitate the labeling of specific markers to predict the risk or the progress of DN and thus facilitating the research on pathological or targeted therapies of DN.

#### 4.2.2 Function and mechanism of m^6^A and methylesterase in T2D

T2D was characterized by classic β-cell dysfunction and IR (1). The considerable current research focused on trying to determine the pathogenic reason for β-cell dysfunction and IR. Recently, scientists revealed the effect of m^6^A methylation on the dysfunction and lower mass of the β-cell, with several proposed mechanisms. The m^6^A modification mediated by the methyltransferases METTL3/14 derived functional maturation of neonatal mouse β-cells, and deletion of METTL3/14 resulted in the inability to establish an adequate amount of functional β-cells after birth (37). A study demonstrated that m^6^A controlled the insulin/IGF1-Akt-PDX1 pathway, and the ablation of m^6^A by targeting METTL3 or METTL14 levels decreased Akt phosphorylation and PDX1 protein levels, resulting in cell-cycle arrest and impaired insulin secretion (15). Another study reported similar results that METTL14 deficiency in β-cell increased cell death, altered cell differentiation, and decreased β-cell mass and insulin secretion (38). The β-cell-specific deletion of METTL3 also induced cell failure and hyperglycemia (39). The mechanism of METTL3/14 regulation of β-cell function was shown to be related to inflammation and oxidative stress (38, 39). Decreased β-cell function can affect insulin secretion and glucose homeostasis. The silencing of FTO expression inhibited insulin secretion by affecting metabolic signaling (29).

In addition, the role of m^6^A modification in IR was also explored in many studies. IR was usually defined as impaired glucose uptake by peripheral tissues and overproduction of hepatic glucose (40). A study found that hyperglycemia enhanced FTO expression in the white blood cells of T2D patients, which promoted the mRNA expression of forkhead box O1 (FOXO1), fatty acid synthetase (FASN), glucose-6-phosphatase catalytic subunit (G6PC), and diacylglycerol O-acyltransferase 2 (DGAT2); all four genes played a critical role in glucose and lipid metabolism *via* IR in T2D (13, 41–44). Another evidence supported that METTL3 and m^6^A were upregulated in the liver tissues of T2D compared with non-diabetes patients, and the silence of METTL3 expression reduced m^6^A methylation and FASN mRNA levels, inhibited fatty acid metabolism thus improving insulin sensitivity, and prevented abnormal lipids and cholesterol metabolism (45). A recent study observed similar results, which reported that the overexpression of METTL3 aggravated high-fat diet (HFD)-induced liver metabolic disorders and IR, the knockout of METTL3 alleviated IR by slowing weight, reducing lipid accumulation (46). METTL3 or METTL14 enhanced the m^6^A methylation of NOD-like receptor protein 3 (NLRP3), which led to the As_2_O_3_-induced hepatic IR (47).

These data not only reflected the vital role of m^6^A and methylesterase in the β cell but also suggested that they were simultaneously involved in IR, which ultimately caused abnormalities in glucose. However, the mechanism is complex and multi-targeted, and more research is still needed to discover its downstream target genes that cause alternation in the corresponding pathways and targets. This will help enrich the understanding of the pathogenesis of DM and provide a theoretical basis for m^6^A-targeted therapy for DM.

#### 4.2.3 Function and mechanism of m^6^A and methylesterase in DN

The development of DN is accompanied by many alternations in the structure of multiple renal compartments, and metabolic changes associated with DM lead to glomerular hypertrophy, glomerulosclerosis, tubulointerstitial inflammation, and fibrosis in the kidney (25). Several studies confirmed that m^6^A and methylesterase were involved in histopathological changes characteristic of DN. For example, the overexpression of METTL14 in HRGECs markedly increased reactive oxygen species (ROS), tumor necrosis factor-alpha (TNF-α), interleukin-6 (IL-6), apoptosis, and suppressed cell proliferation by suppressing the m^6^A modification of α-klotho, while α-klotho can prevent tubular and glomerular injury and delayed DN (35, 48). However, another study revealed that podocyte-specific METTL14 deletion upregulated Sirt1 expression, thereby alleviating apoptosis and inflammation, regulating autophagy, and delaying the development of proteinuria and glomerulosclerosis (34). Another study reported the overexpression of METTL14 reversed high-glucose-activated phosphatidylinositol-3-kinase/protein kinase B(P13K/Akt) pathway inactivation in HK2 by enhancing phosphatase and tensin homolog (PTEN), followed by the downregulation of histone deacetylase 5 (HDAC5), thus ameliorating DN manifestations such as fibrosis, inflammation, cell death, and albuminuria (32, 49). Overexpression of METTL3 enhanced the stability and expression of nuclear receptor-binding SET domain protein 2 (NSD2) in the high-glucose-induced mouse mesangial cell lines and that NSD2 overexpression attenuated pathological changes in the kidney, including glomerular dilatation, glomerulosclerosis, thylakoid proliferation, and interstitial fibrosis (31). METTL3 was highly expressed in the podocytes of db/db mice and streptozotocin-induced mice compared with db/m mice, and the upregulated expression of METTL3 in podocytes induced by high glucose accounted for the aberrant m^6^A modification. The METTL3-mediated m6A modification level of TIMP2 mRNA may promote podocyte injury, apoptosis in glomeruli, and kidney inflammation through upregulating the Notch3 and Notch 4 signaling pathways (30). The knockdown of WTAP inhibited pyroptosis and diminished the release of IL-18, IL-1β, caspase-1, and NLRP3 in high-glucose-induced HK-2 cells (33). In clinical applications of regulators targeting RNA modification enzymes, a cell experiment demonstrated the total flavones of Abelmoschus Manihot, the main components of the Huangkui capsule and ameliorated pyroptosis and injury in podocytes under high-glucose conditions by adjusting METTL3-dependent m^6^A modification and regulating NLRP3-inflammasome activation and PTEN/PK13/Akt signaling (50).

Methylatase-mediated m^6^A modifications were involved in various renal tissue structural and functional alternations in DN. However, the mechanisms were intricate and complex, covering multiple targets and multiple pathways in various cells. Therefore, future research is necessary to investigate which methylatases are involved in the pathogenesis of DN and determine the exact regulatory and functional mechanisms of these methylatases that participated in the development and progression of DN, and if possible, mechanistic studies on different periods of DN and continuous follow-up may provide a new target for the prevention or treatment of DN.

In this article, more RNA methylation studies focused on T2D and DN, with a small number of studies revealing the role of m^6^A methylation in other complications of DM. Diabetic retinopathy is the main cause of visual disability and blindness in DM (51). A recent study suggested that alteration of m^6^A was related to the pathogenesis of diabetic retinopathy such as inflammation, oxidative, and angiogenesis (52). The overexpression of METTL3 alleviated high-glucose-induced retinal pigment epithelium cell division, apoptosis, and pyroptosis, and reduced the levels of IL-1β and IL-18. The overexpression of METTL3 promoted cell proliferation by regulating miR-25-3p/PTEN/Akt signaling (53). However, in another study, the level of m^6^A RNA modification and METTL3 was increased in diabetic retinal vessels, and the increased METTL3 accelerated pericyte apoptosis and decreased pericyte viability (54). Based on the current research baseline and research background, RNA methylation may be a new hotspot and direction for research on other complications of DM and types of DM.

#### 4.2.4 Risk factors for CVD in DM and its complications

There were few studies of RNA methylation in DM combined with CVD, but in this article, the keywords “cardiovascular disease”, “risk factors”, “mellitus”, and “m^6^A” appeared both in the burst detection of keywords and references. It is known that T2D is an independent risk factor for CVD; hyperglycemia, IR, dyslipidemia, inflammation, and oxidative stress were also associated with CVD in DM (17, 23). The mechanisms of the above risk factors were all associated with m^6^A methylation and methylesterase.

Emerging evidence indicated that m^6^A was closely related to the occurrence and progression of CVD (9, 17). However, whether m^6^A modification is involved with CVD in DM remained largely incomplete. Diabetic cardiomyopathy (DCM) is defined as impairments of cardiac structure and function caused by the dysregulated glucose and lipid metabolism associated with DM (55, 56). A study demonstrated that in the diabetic heart disease mice model, FTO was downregulated in the heart tissue, and the overexpression of FTO improved the cardiac function by reducing myocardial fibrosis and myocyte hypertrophy in db/db mice (57). METTL14 was significantly downregulated in the heart tissue and serum samples of DCM rats compared to those of normal rats (58). Pyroptosis is programmed cell death and the consequent release of pro-inflammatory mediators such as caspase-1. Several studies suggested that pyroptosis played an important role in the progression of DCM (59). Enhanced METTL14 inhibited pyroptosis levels in myocardial tissues, including the downregulation of NLRP3, caspase-1, and gasdermin D through downregulating the expression of TINCR lncRNA and NLRP3 (58, 60, 61). The altered cardiac metabolic pathways were also involved in the pathogenesis of DCM (55, 62). A recent study demonstrated that FTO regulates glycolysis in an m^6^A-dependent manner and also regulated glucose metabolism by modulating the Akt-GLUT4 axis in the heart failure mouse model, thus regulating the energy supply in the cardiac function and structure (63). Lipotoxicity was also evident in cardiac metabolism. A study demonstrated that FTO not only facilitated adipogenesis and lipid droplet formation but also disordered lipid utilization in skeletal muscles through the inhibition of PPARβ/δ and AMPK pathways; the upregulation of FTO also reduced insulin secretion by the inflammatory NF-κB pathway and led to the development of hyperglycemia and hyperlipidemia (64). Moreover, the other mechanisms of DCM referred to in the Discussion section such as impaired insulin sensitivity, inflammation, and oxidative stress were also closely connected with the progression of DCM (55, 56). However, there were few direct studies that showed a relationship between RNA methylation and other mechanisms of DCM. Therefore, we supposed that methylesterase-mediated m^6^A modifications may play a bridge role between the mechanism of DCM and DM. The m^6^A methylesterase may be a risk forecast indicator for CVD in DM, but further studies are warranted to systematically assess the role of m^6^A modification and the change of m^6^A methylesterase in DM combined with CVD.

Apart from the most studied and most abundant m^6^A RNA modification, some other types of RNA methylation have been identified, such as N1-methyladenosine (m^1^A), 5-methylcytosine (m^5^C), N3-methylcytosine (m^3^C), N7-methylguanosine (m^7^G), 2′-O-methylation, etc., and many studies also revealed their function and mechanism for various diseases (65–70). However, relatively little literature was collected in this paper, although other forms of RNA methylation were retrieved in the Web of Science Core Collection. This may be caused by the fact that other RNA methylation may not have been studied or published yet in DM and its complications, which indicates that we are still at the threshold of this new frontier research that may provide a new research direction in DM and its complications.

## 5 Strengths and limitations

This study is the first bibliometric analysis to systematically analyze publications related to RNA methylation in diabetes mellitus and its complications in the past 20 years. Unlike traditional systematic reviews, the bibliometric analysis objectively and comprehensively quantifies and evolutionize research hotspots and trends in a field by mathematical techniques. In this review, not only the evidence of hotspots and trends in RNA methylation of DM and its complications were objectively visualized but also the research on the current achievements and prospects was systematically summarized. Moreover, according to the results of the hotspots and frontiers, we provided a detailed review of the change and the mechanisms of m^6^A and methylesterase in T2D and DN. We also summarized the mechanism of m^6^A and methylesterase in DCM. It is hoped that the comprehensive picture of RNA methylation especially m^6^A modification in this review will serve as a baseline and guide for the future development of DM and its complications. Meanwhile, it is hoped that the summary of the current research helps researchers quickly identify the strengths and weaknesses and thus enrich and improve the development of the field.

Inevitably, there were some limitations in this study. Firstly, the restricted database and time make retrieved literature incomplete. Only representative Web of Science Core Collection databases were searched and were limited to the period of 1 January 2002 to 28 May 2022; some updated published literature was not included. Secondly, the diversity of subject terms or the incompleteness of the literature reduced the credibility of atlas mapping. Finally, incomplete extraction of a few isolated keywords by software, articles containing incomplete items excluded by software, or a deficiency in the research itself may also affect the accuracy of the results.

To minimize limitations, we additionally manually retrieved fewer keywords and the updated article and summarized them with the results of the bibliometric analysis to provide scholars with the most recent comprehensive reviews, to quickly get the research backgrounds and keep up with the hotspots and trends in RNA methylation of DM and its complications.

## 6 Conclusion and perspectives

This reversible RNA methylation added a new dimension to the development of post-transcriptional regulation of gene expression. Convincing evidence suggested that m^6^A modification provided novel substantial perspectives on the physiopathology of DM and its complications. In this field, Canada, the USA, and China published the most articles; Univ Toronto and St Michaels Hosp were the leaders in the publication; and professors such as Leiter and Sievenpiper have made outstanding contributions in this field. The literature related to RNA methylation has received the attention of high-level journals and has been widely cited. Especially, m^6^A writers, readers, and erasers were the hotspots and trends in T2D and DN, as recent advances highlighted their contribution to the numerous physiological processes of cells and diverse pathological mechanisms of DM and its complications. However, looking toward the future, there are still many significant knowledge gaps to be completed. Firstly, strengthening national and institutional interactions and collaboration is necessary to produce more achievements for the positive upward and rapid expansion of this field. Secondly, the m^6^A modification had a difference in organisms under different conditions, reflecting its complex multi-pathway and multi-target mechanism, and DM also causes multi-organ and tissue damage. Much consolidation evidence is still needed to explore its intricate network mechanisms. Also, other RNA modifications, including m^1^A and m^5^C, should be further explored. Thirdly, much of the current research has been confined to molecular mechanisms; more attention is urgent, albeit difficult and protracted, and needs to be paid to the clinical applications targeting m^6^A, such as proposing non-invasive clinical specific biomarkers of the mechanisms or progression of DM, especially predictive markers of risk for its complications DN and CVD. Meanwhile, research on small-molecule modulators targeting m^6^A for DM and its complications is necessary to fill the gaps in current clinical applications.

## Author contributions

YG, MZ, and WZ conceived the work. WZ wrote the manuscript. YG and MZ discussed and edited the manuscript. SZ, CD, and SG collected and analyzed the data. WJ, YJ, and CW checked the results. All authors contributed to the article and approved the submitted version.

## Funding

This work was supported by the National Administration of Traditional Chinese Medicine Young Qi Huang Scholars support project (National Traditional Chinese Medicine Human Education Development [2020] No. 7).

## Acknowledgments

The authors thank Dongzhimen Hospital, Beijing University of Chinese Medicine, and Beijing Hospital of Traditional Chinese Medicine, for their support of this work and the reviewers for allowing us to improve the manuscript.

## Conflict of interest

The authors declare that the research was conducted in the absence of any commercial or financial relationships that could be construed as a potential conflict of interest.

## Publisher’s note

All claims expressed in this article are solely those of the authors and do not necessarily represent those of their affiliated organizations, or those of the publisher, the editors and the reviewers. Any product that may be evaluated in this article, or claim that may be made by its manufacturer, is not guaranteed or endorsed by the publisher.
