# Supplementary material for: A bibliometric analysis of RNA methylation in diabetes mellitus and its complications from 2002 to 2022
Source: Front Endocrinol (Lausanne). 2022 Sep 8;13:997034. doi: 10.3389/fendo.2022.997034 (PMC9492860; doi:10.3389/fendo.2022.997034)
Supplement: Supplementary file 1 [file Table_1.docx]

Supplementary Table 1 | The search queries of WOS related to RNA methylation in DM and its complications

| No. | Query |
| --- | --- |
| 1 | ((((((((((TS= (diabetes mellitus)) OR TS=(diabetes)) OR TS=(diabetic)) OR TS=(diabetics)) OR TS= (diabetic mellitus)) OR TS= (diabetics mellitus)) OR TS= (type 2 diabetes mellitus)) OR TS= (type 1 diabetes mellitus)) OR TS= (gestational diabetes mellitus)) OR TS= (aged diabetics)) OR TS= (diabetes patients) |
| 2 | (((TS=(hyperglycemia)) OR TS= (high glucose)) OR TS=(hyperglycemic)) OR TS=(Glycemic) |
| 3 | (((((TS= (diabetic nephropathy)) OR TS= (diabetic nephropathies)) OR TS= (diabetic kidney disease)) OR TS= (diabetic nephropathy patients)) OR TS=(diabetes nephropathy)) OR TS=(diabetic nephrosis) |
| 4 | (TS= (diabetic retinopathy)) OR TS= (diabetes retinopathy) |
| 5 | (((TS= (diabetic peripheral neuropathy)) OR TS= (peripheral neuropathy)) OR TS= (diabetic neuropathy)) OR TS= (Diabetic Nephropathies) |
| 6 | (TS= (diabetic foot)) OR TS= (diabetes foot) |
| 7 | (ALL= ("RNA Methylation")) |
| 8 | ((((((((ALL=(m^6^A)) OR ALL= ("m (6)A")) OR ALL=(m^6^A modification)) OR ALL=(N6-methyladenosine)) OR ALL=(N-6-methyladenosine)) OR ALL=("adenosine N6 methylation")) OR ALL=(6-methyladenine)) OR ALL=(m6A methylation)) NOT ALL= ("DNA Methylation") |
| 9 | ((((((((ALL=(m1a)) OR ALL=(ac4c)) OR ALL=(m5c)) OR ALL=(m7G)) OR ALL=(pseudouridylation)) OR ALL=(2’-O-methylation)) OR ALL=(2’-O-Me)) OR ALL=(2’-O-RNA)) OR ALL=(U-trail) |
| 10 | #1 OR #2 OR #3 OR #4 OR #5 OR #6 |
| 11 | #7 OR #8 OR #9 |
| 12 | #10 AND #11 |

Supplementary Table 2 | The top 10 productive institutions related to RNA methylation in DM and its complications.

| No. | Institution | Location | Count | Centrality |
| --- | --- | --- | --- | --- |
| 1 | Univ Toronto | Canada | 205 | 0.6 |
| 2 | St Michaels Hosp | Canada | 154 | 0.18 |
| 3 | McMaster Univ | Canada | 31 | 0.01 |
| 4 | Univ Saskatchewan | Canada | 28 | 0.02 |
| 5 | Univ Melbourne | Australia | 15 | 0.01 |
| 6 | Univ British Columbia | Canada | 14 | 0 |
| 7 | Univ Hlth Network | Canada | 13 | 0.01 |
| 8 | Texas A&M Univ | USA | 11 | 0.11 |
| 9 | Inst Clin Evaluate Sci | Canada | 10 | 0 |
| 10 | Harvard Med Sch | USA | 10 | 0.01 |

Supplementary Table 3 | The top 8 authors and cited authors related to RNA methylation in DM and its complications.

| No. | Author | Count | Location | Cited author | Citation |
| --- | --- | --- | --- | --- | --- |
| 1 | Leiter LA | 44 | Canada | Jenkins DJA | 55 |
| 2 | Sievenpiper JL | 30 | Canada | Wolever TMS | 51 |
| 3 | Kendall CWC | 30 | Canada | Wang X | 41 |
| 4 | Gilbert RE | 29 | Canada | Sievenpiper JL | 30 |
| 5 | Wolever TMS | 27 | Canada | Meyer KD | 28 |
| 6 | Jenkins DJA | 24 | Canada | Jia GF | 28 |
| 7 | Advani A | 24 | Canada | Yang Y | 25 |
| 8 | Jenkins AL | 16 | Canada | Dominissini D | 24 |
| 9 | Kelly DJ | 13 | Australia | Advani A | 21 |
